# Design and Manufacturing of Experimental Solid Propellant Rocket Motor Cases Made of Carbon Composite Materials

**DOI:** 10.3390/polym17101352

**Published:** 2025-05-15

**Authors:** Berdiyar Baiserikov, Marat Ismailov, Laura Mustafa, Nurmakhan Yesbolov, Arman Kulbekov, Abussaid Yermekov, Mohammed Meiirbekov, Ilyas Ablakatov

**Affiliations:** JSC National Center of Space Research and Technology, Almaty 050010, Kazakhstan; baiserik.b.91@mail.ru (B.B.); m.ismailov@gmail.com (M.I.); nurmahan_esbolov@mail.ru (N.Y.); a.kulbekov@spaceres.kz (A.K.); a.yermekov@spaceres.kz (A.Y.); m.meiirbekov@spaceres.kz (M.M.); ablakatov.i@spaceres.kz (I.A.)

**Keywords:** polymer composite material, epoxy resin, carbon fiber, strength, solid propellant rocket motor, filament winding

## Abstract

This paper investigates a polymer composite and carbon fiber impregnated with epoxy resin for the fabrication of a lightweight and high-strength composite casing for rocket propulsion systems. It describes the winding technology which uses a removable mandrel and angular winding at ±55° and ±20° to expand the stress distribution, as well as alternating angles of ±45° and 80° to improve resistance to tensile and torsional loads. A fixture has been developed that ensures ease of disassembly and good strength of the final products. Hydrostatic tests showed the operational stability of the casings under internal pressure up to 10 MPa for a 1.5 mm-thick casing and 18 MPa for a 3 mm-thick casing, which confirms the effectiveness of the proposed technology. The research results demonstrate the high reliability and potential exploitation of composite materials.

## 1. Introduction

Rocket design must ensure precise subsystem placement, resilience to random effects, and maximum strength-to-weight ratio to meet mission requirements. Advances in structural design and analysis are contributing to the performance of aerospace systems in a variety of environments. The solid propellant rocket motor (SRM) casing is a key component that essentially affects the rocket’s flight characteristics, requiring an optimized design to improve mission performance [1]. It is manufactured from composite materials due to its high strength, low weight, and resistance to extreme thermal stresses. With the introduction of composite materials such as carbon and glass fibers, the weight and strength characteristics of the casings have been significantly enhanced, making them attractive for aerospace applications [2,3].

As is proven in practice, one of the most widely used and effective methods of composite vessel manufacturing is filament winding. The filament winding method consists of impregnating carbon, glass, or other types of fibers with thermosetting resin and layering them on a rotating mold (mandrel). This process is carried out with high precision and control over the orientation of the fibers to achieve optimum mechanical properties for the finished product. The enclosure structure itself is created in multiple layers, and each layer can be wound at a specific angle to maximize strength and resistance to deformation and stress. Enclosures are produced from glass fiber or organic or carbon filaments [4].

To date, the application of carbon fiber-reinforced composite materials in the aerospace industry is well established due to their high stiffness and strength with low specific gravity compared to metal alloys. The major approaches in the development of technology for manufacturing SRM casings from high-strength carbon fibers are based on vessel design, selection of optimal materials, efficient tooling, lay-up technologies, and quality control methods to ensure high mechanical properties and durability of the structure under conditions of substantial loads and temperatures. The combination of epoxy resin matrix composites with carbon fibers provides a high level of strength and resistance to thermal and mechanical loads [5,6].

Whilst designing the technology for manufacturing SRM casings, the key aspects are optimization of winding parameters, modification of structural characteristics, and reduction in residual stresses in the finished product. For this purpose, the scientific literature suggests approaches that take into account the case geometry, mandrel materials, and the layered structure of composites. According to the results of a review paper [1], the highest mechanical properties of SRM housings are achieved at winding angles in the range from 52.1° to 55° [6,7]. This value is optimal to provide a balance between axial and circumferential stiffness. The analysis of [3] showed that a winding angle of ±54.7° provides a uniform stress distribution at internal pressures typical of SRM operation.

However, for complex body shapes and different loads, adaptive variation in winding angles is recommended. For example, combining annular and spiral layers can enhance the stability of the casing under axial and radial loads [8]. This is especially relevant for the cylinder–dome interface zones, where the maximum stresses may reach critical values. Studies [8] show that alternating layers at angles of 0°, ±45°, and 90° provide the best resistance to mechanical loads.

The authors of [9] put emphasis on multiscale modeling that considers the parameters of winding angles and thickness of composite layers. Mesoscale and microscale analysis showed that matrix failure, interlayer damage, and fiber failure occurred in different zones of the body under different loads. At a pressure of 10 MPa, the damage initiated in the cylinder and transition zone, whilst at 30 MPa, the failure region expanded, and the fibers were completely destroyed in the transition zone. Research shows [1,10] that optimizing the thickness of each layer of composite material plays a crucial factor in minimizing residual stress and preventing interlayer damage. Improper thicknesses result in stress concentration zones, especially in the transition regions between the dome and the body cylinder.

The authors of [2] investigated the behavior of a cylindrical skirt under internal pressure and axial thrust by comparing the performance of skirts made of steel and carbon fiber-reinforced composite material. The composite exhibited high tensile strength (1.47 GPa) and compressive strength (0.98 GPa), showing an excellent strength-to-weight ratio. Stability analysis indicated that the composite could withstand loads up to five times the operational loads. A study by [11] revealed that the combination of T-700 carbon fiber with LY 556 and LY 5052 hybrid resin at a 50:50 ratio improves the tensile strength to 1927.33 MPa. ANSYS finite element numerical analysis results confirm that the enclosure withstands an internal pressure of up to 105 bar and a critical axial load of 250 kN without fracture.

As follows from the analysis of the literature data, researchers are actively engaged in the development of SRM casings, devoting special attention to the development of materials with optimal properties to ensure their strength, reliability, and durability. Nevertheless, for further enhancements of technologies of design and manufacturing of SRM vessels, in-depth studies aimed at precise control of winding parameters are required. The scientific interest of this work lies in the study of overlooked stacking angles and the application of modified epoxy resins, which opens up new opportunities for improving SRM casing materials with advanced properties.

## 2. Materials and Methodology

### 2.1. Materials

Epoxy resin (ER) was used as the matrix material of the SRM composite body, which consisted of the epoxy resin Ethal Inject T itself (JSC Epital, Moscow, Russia) and plasticizer tricresyl phosphate (Zhangjiagang Fortune Chemical Co., Ltd., Zhangjiagang, China). The choice of ER is justified by the fact that it provides fast and high-quality impregnation of glass and carbon fibers due to its low viscosity. The finished composites show high strength properties, water resistance, and alkali resistance. The injection of tricresyl phosphate (TCP) into the matrix gives an increase in the strength properties of the final composite [12,13,14,15,16]. Table 1 and Table 2 summarize the general characteristics of ER and TCP.

**Figure 1:**
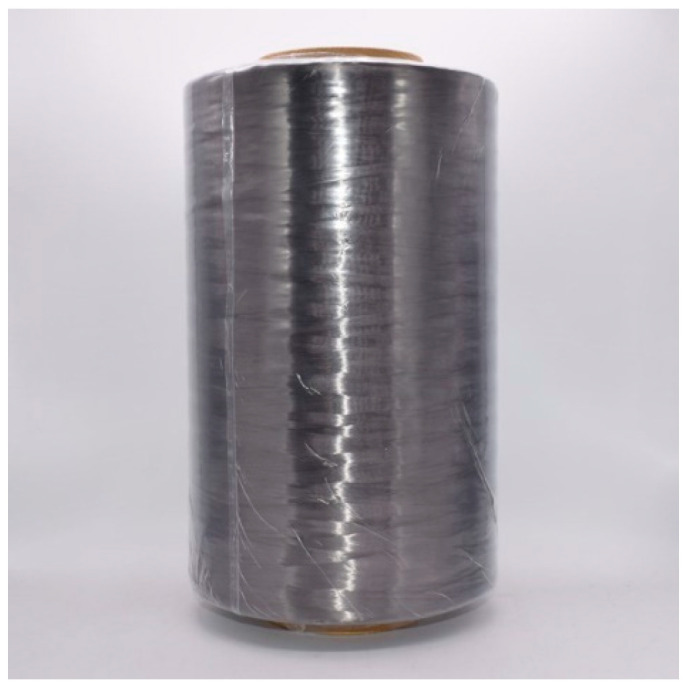
Carbon roving TORAY T700SC, 12k.

Carbon roving made of high-strength carbon fiber TORAY T700SC 12k (Toray Composite Materials America, Inc., Tacoma, WA, USA) was used as a reinforcing material of the vessel. The appearance of carbon roving (CR) is presented in Figure 1. UR Toray T700S 12k, due to high temperature resistance, lightness, and mechanical strength, is used extensively in aerospace and aviation. The main characteristics are given in Table 3.

Structural steel 30 KhGSA (OOO MetallSklad Severa-Zapad, Sankt-Peterburg, Russia manufacturer, GOST 4543-71) was utilized for fabrication of the body flanges. The advantages of steel 30 KhGSA are as follows: high tensile strength 1700 MPa; high hardness 50 HRC; wear resistance; resistance to variable loads; excellent weldability; and high resistance to constant thermal effect (up to 400 °C) [17].

### 2.2. Methods

An MSH-300 magnetic stirrer (BioSan, Rīga, Latvia) heated to +330 °C, up to 15 L and 1250 rpm, was exploited to mix ER with hardener and plasticizer at an additional 80 °C heating.

**Figure 2:**
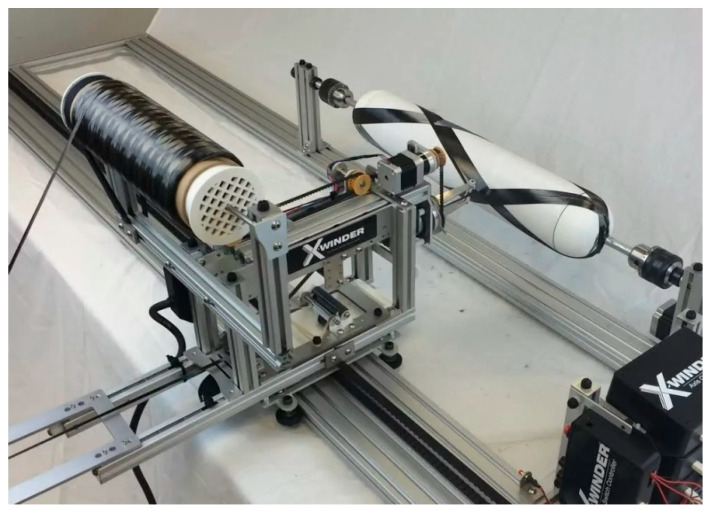
Case winding machine “X-Winder”.

A small-sized 4-axis X-Winder winder (Figure 2) with software control was used for winding the body, which allows winding products up to 3000 mm in length and 200 mm in diameter.

For hydrostatic tests on strength and tightness of the hull, the high-pressure unit Poseidon B24 (OOO Zet-Tekhno, Moscow, Russia) was used, which is produced on the basis of a petrol engine and a reliable high-pressure pump. For operation in conditions of water shortage, the machinery is equipped with water tanks of 200–1000 L capacity and a booster pump.

## 3. Results

### 3.1. Design of a Lightweight, High-Strength Composite SRM Casing

**Figure 3:**
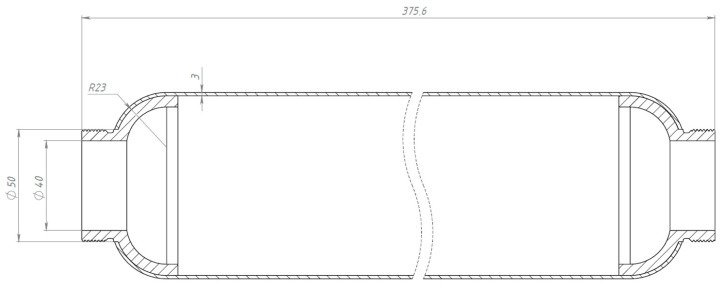
General view of a drawing of a composite casing with a power shell wall thickness of 3 mm.

A lightweight, high-strength composite SRM casing is designed. For the design, a non-displaceable enclosure of the «cocoon» type, made of high-strength carbon fiber-reinforced plastic by wet winding method, was determined. This design provides an exceptional weight-to-strength ratio, simplifies the manufacturing process, and guarantees equal strength of the product [18,19]. The cocoon-type enclosure comprises the following key elements: power enclosure shell (PES); front flange; rear flange; front joint assembly; and rear joint assembly. The PES supports the internal pressure and external force loads acting on the casing. Figure 3 shows the general view of the designed SRM composite casing.

The designed model of the enclosure has the shape of a cylinder with an inner cavity with end hemispherical bottoms, which allows us to reduce the weight of the component without essential loss of strength. The length of the cylindrical part of the enclosure is 300 mm. The structure consists of several layers of composite, each with a differing fiber orientation for optimum stress distribution and strain resistance (fiber orientation is given in the Results Section). The design is adapted for conditions where resistance to longitudinal and radial loads is required. The front flange is used to attach the cover with the ignition device. The rear flange is used to mount the nozzle assembly. The metal flanges were wound into the body during the formation of the power casing.

Strength calculations of the designed composite casing model were carried out in ANSYS software (2024R1). Fixed supports are used to simulate the fixation of the structure in real conditions. Ultimate loads occurring during engine operation are applied on the inner surface of the cylinder.

**Figure 4:**
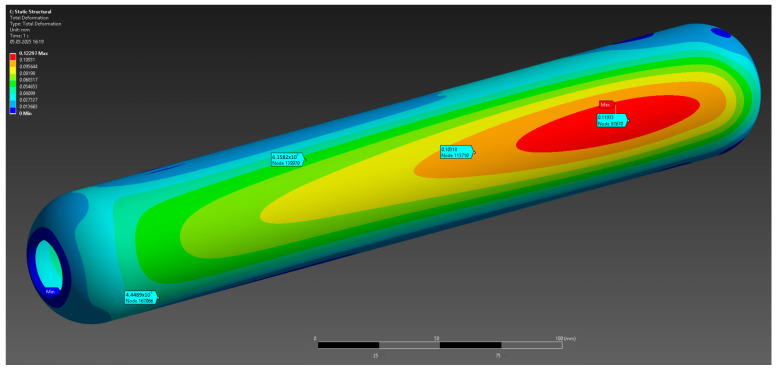
Total deformation.

**Figure 5:**
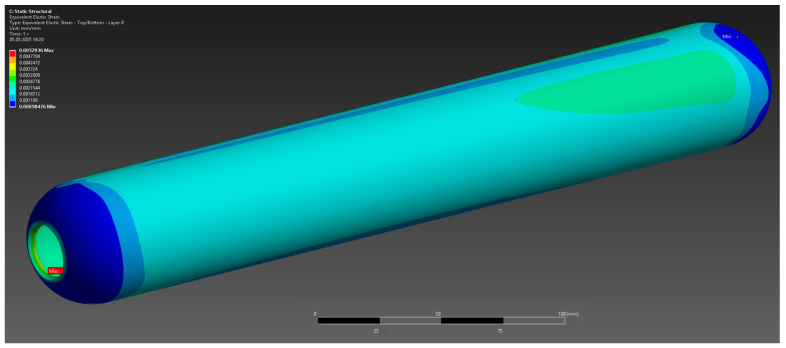
Elastic deformation.

**Figure 6:**
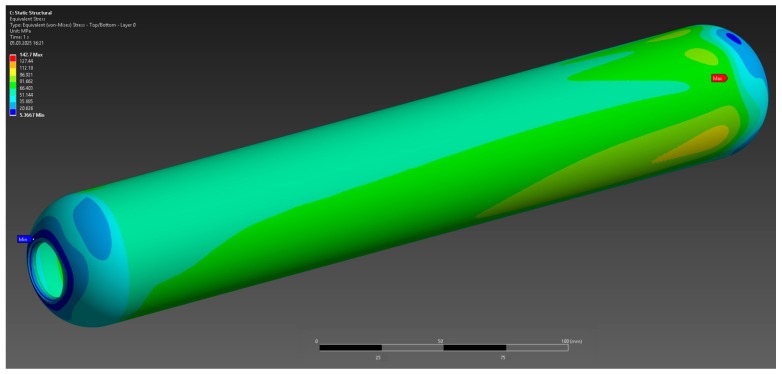
Equivalent strain limit.

The results of the numerical analysis demonstrated that the maximum observed deformation of the casing is 0.1229 mm, with the highest values recorded near the free end, away from the fixed supports. This value is within acceptable limits, confirming the preservation of the structural integrity of the casing at a given pressure of 10 MPa (Figure 4). The maximum equivalent elastic strain was 0.0052 mm/mm (Figure 5). The areas of high stress were concentrated near the inner surfaces of the hollow section and around the fixed supports, which is expected under these loading and anchorage conditions. The maximum equivalent Mises stress reached 142 MPa, with the areas of highest stress coinciding with the areas of maximum strain, which allows the identification of the critical points of the structure (Figure 6). The stress values are below the yield strength of the material, which indicates the presence of a sufficient safety margin of the casing at operating pressure.

The safety margin was calculated according to the ultimate strength of the material and the highest stress arising in the digital model. For SRM casing, the permissible safety margin is taken as *n* ≥ 1. The ultimate strength of the composite material is 425 MPa, and the maximum stress in the case is 142 MPa. The safety margin of the structure can be calculated by the formula [20]:

(1) $$n=\frac{\sigma_{B}}{\sigma }\geq \left[n_{min}\right]=\frac{425}{142}=3\geq 1.5$$

where $\sigma_{B}$—material tensile strength, and σ—peak stress.

The conducted analysis confirms the compliance of the design with the specified requirements of the safety factor *n* = 3, which is well above *n* = 1. The mass of the composite power casing of the designed enclosure was m = 497 g.

**Figure 7:**
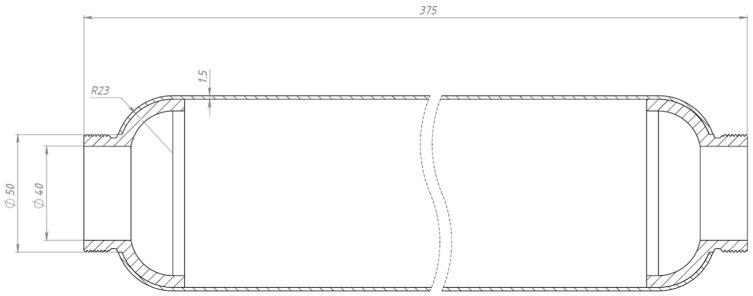
General view of the drawing of the composite case with a wall thickness of 1.5 mm of the power shell.

In consideration of the high design safety factor *n* = 3, in order to optimize and lighten the structure, the engine casing was designed with a smaller wall thickness of the casing power shell, 1.5 mm (Figure 7). Similar strength calculations were performed for the motor casing with a wall thickness of 1.5 mm.

**Figure 8:**
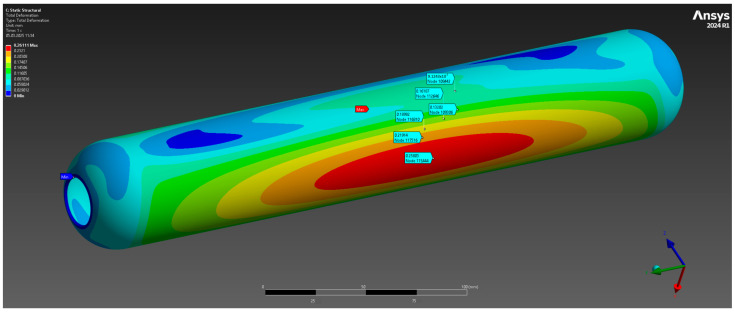
Total deformation.

**Figure 9:**
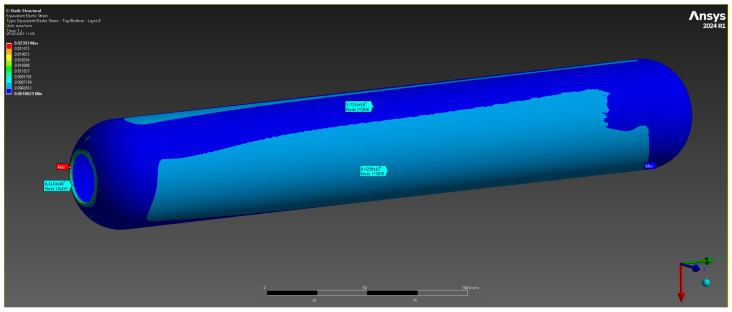
Elastic deformation.

**Figure 10:**
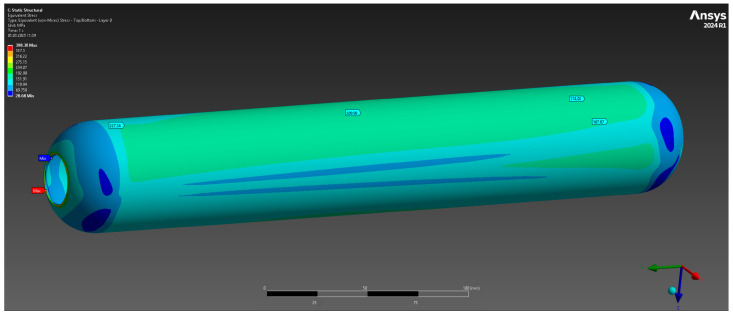
Equivalent strain limit.

The results of numerical modeling demonstrated that the maximum deformation of the casing is 0.26 mm, which is within the acceptable values and confirms the preservation of structural integrity at an internal pressure of 10 MPa (Figure 8). The maximum equivalent elastic strain reached 0.024 mm/mm (Figure 9). Zones with increased stress are localized near the internal surfaces of the hollow section and anchorage areas, which is consistent with the expected load distribution. The maximum equivalent Mises stress was 398 MPa, with critical stresses observed in the same areas as the maximum strain (Figure 10). This indicates potential vulnerable points in the structure which were identified and considered in the design.

The stress values are below the yield strength of the material, demonstrating a sufficient safety margin for the enclosure under the specified operating conditions. Safety margin:

(2) $$n=\frac{\sigma_{B}}{\sigma }\geq \left[n_{min}\right]=\frac{425}{398}=1.067$$

The conducted analysis confirms the compliance of the design with the given requirements of the safety factor *n* = 1.067. The mass of the composite power casing of the designed enclosure totaled m = 242 g.

Thus, lightweight, high-strength composite SRM casings with a safety factor *n* ≥ 1 at an internal pressure of 10 MPa were designed for the development of manufacturing technologies.

According to the results of the analysis, the type of collapsible metal mandrel, which will ensure stability and accuracy in the process of winding [21], was selected to develop the technology of winding the composite SRM casing.

Thereby, lightweight, high-strength composite SRM casings with a safety factor *n* ≥ 1 at an internal pressure of 10 MPa were designed. The analysis of strength characteristics in ANSYS revealed the potential for optimization of the design with a wall thickness of the power casing up to 1.5 mm. The optimization reduced the mass of the power casing from 497 to 242 g whilst maintaining the required strength of the structure. The results of computer modeling confirmed the performance of both variants of the casing under the defined operating conditions.

### 3.2. Design and Fabrication of Winding Process Tooling

The technological mandrels used for winding composite SRM casings are the core elements that determine the shape and structure of the final product. Mandrels serve as a base on which reinforcing fiber layers are placed with the necessary angle and tension to obtain the required mechanical properties [22].

**Figure 11:**
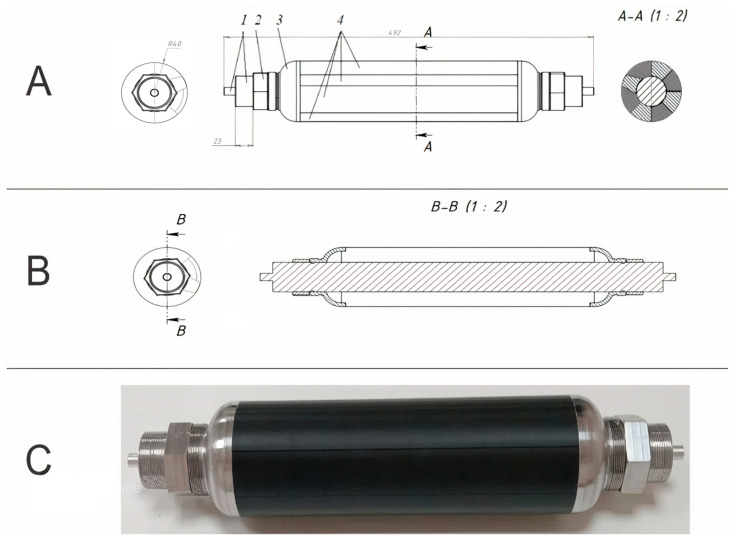
Close-up view of the drawing of the collapsible tooling (**A**), sectional view of the drawing (**B**), the general view of the assembled removable mandrel (**C**). and a snapshot thereof, where 1—center shaft; 2—nut for assembly of base parts; 3—flange; 4—cylindrical part of tooling (segments).

In this work, a technological collapsible tooling for winding the composite SRM body has been developed, the parameters of which are illustrated in Figure 11. The tooling has a length of 492 mm, a diameter of the cylindrical part of 80 mm, and a diameter of the flange bore of 50 mm. The design of the tooling conforms to the geometry and shape of the inner surface of the designed case, ensuring easy removal after curing, as well as a smooth and even surface. The principal task in the design was to provide increased manufacturability of mandrel fabrication and dismantling, minimize its weight, and ensure high load-bearing capacity of the wound casings.

The designed drawings were used to manufacture the metal parts of the mandrel: fastening nuts, metal flanges for the pole bottoms of the casing, and the central assembly shaft. The metal parts were fabricated by machining on a numerically controlled machine CK42FY (Jiangsu CKE Machine Tool Co., Ltd., Tientsin, China manufacturer). To facilitate the construction, the segments of the cylindrical part of the tooling are made of plastic by 3D printing FLSun V400 (Zhengzhou Chaokuo Electronic Technology Co., Ltd., Zhengzhou, China).

The collapsible mandrel design consists of three parts: insert flanges profiled for the body bottoms; a cylindrical shell consisting of eight collapsible segments (one of which has an inverted geometry for precise positioning); and a central shaft with mounting nuts. The mandrel parts forming the bases are mounted in the shaft slots, after which the cylindrical segments are installed. Fastening of all elements is carried out by means of fastening nuts, which ensures the strength of the structure during winding. The composite material was wound onto the assembled mandrel to form the SRM body (Figure 11C).

The technological tooling for winding of the composite SRM body has been designed and manufactured, providing high geometric accuracy, and processability of assembly and disassembly. The design of the tooling is optimized by using multiple materials: the flanges are made of 30 KhGSA steel, which provides strength and durability; the shaft is made of D16 aluminum alloy, which contributes to reducing the weight of the structure; and the segments of the cylindrical part are made of PLA plastic by 3D printing, which additionally reduces weight and simplifies dismantling. The fabricated tooling fully complies with the requirements of the technological process of winding the composite SRM casing.

### 3.3. Development of Technology for Manufacturing SRM Case Power Shell from High-Strength Fibers Applying Combined Reinforcement

Technological development is an essential stage that determines the success of the entire process, since it is at this point that the fundamental parameters of the design are established. The stacking angle and layer thickness during winding of the SRM case require particular attention, as they dramatically affect the stress distribution, strength characteristics, and durability of the product. Also, the use of combined stacking angles helps to improve the mechanical properties of the SRM casing, providing optimal load distribution and increased resistance to multiple types of deformations [23,24,25].

The SRM power shell (PS) manufacturing process was developed by winding high-strength carbon fibers impregnated with epoxy resin onto collapsible tooling using combined lay-up angles. The process was performed on an ‘X-Winder’ 4-axis winding machine, which provides high accuracy and repeatability. The X-Winder software V420 4.2 allowed the winding parameters to be set, including lay type and pattern, roving size, tooling geometry, and operating mode. For the cylindrical shape with hemispherical ends, a 4-axis winding type was chosen to ensure an optimal fiber distribution and high mechanical strength. The fibers were passed through a vessel filled with epoxy resin to ensure uniform saturation with the matrix. The feed carriage moved along the tooling according to a predetermined algorithm, providing the required winding mode and minimizing the probability of defects. The winding algorithm was calculated taking into account the geometrical features of the tooling, which allowed us to achieve uniform stacking and optimum mechanical characteristics of PS.

Using the method of continuous fiber winding on tooling with alternating ring and spiral lay-up, casings with wall thicknesses of 1.5 mm and 3 mm were fabricated. The optimized winding angles of ±55° and ±20° ensured uniform stress distribution, which is particularly important for cylindrical casing segments. For hemispherical and cylindrical segments, alternating angles of ±45° and 80° were utilized, which considerably improved the structure’s resistance to tensile and torque loads. This approach also kept deformations under internal pressures to a minimum, ensuring high strength and stability of the casings under operating loads.

**Figure 12:**
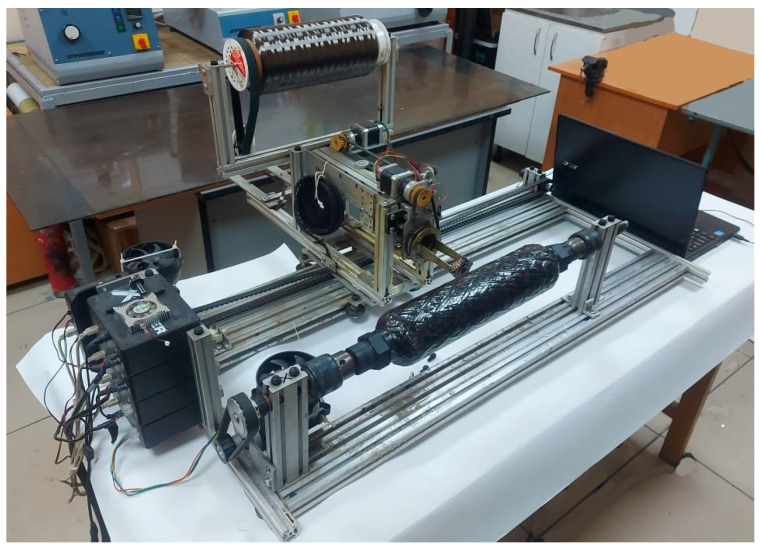
A wound composite case.

Figure 12 shows a wound composite case. The fabrication of the enclosure went through the following steps:

- Preparation of the mandrel for winding;
- Preparation of the winding binder: modified epoxy compound Ethal Inject T;
- Filling the impregnation bath with binder;
- Preparation of carbon roving: drying of the roving roll at 100 °C for 2 h;
- Installation of the roving roll into the impregnation line;
- In X-Winder software V420 4.2, setting the necessary winding parameters: winding angles, layers of angles, roving parameters;
- Winding the casing at the specified laying angles;
- Finalizing the winding and extracting the finished cured casing.

The thickness of one winding layer equaled 0.25 mm. To form a motor casing with a total wall thickness of 3 mm, an equal number of layers were applied at varying lay-up angles: 80°, 55°, 20°, and 45°—three layers of each. For the 1.5 mm wall thickness case, the required number of layers varied: 80°—two layers, 55°—one layer, 20°—one layer, and 45°—two layers. Curing of the composite body was carried out at room temperature for 24 h, which ensured complete formation and stabilization of the material structure.

After curing the SRM casing, the mandrel disassembly process was accomplished in the following sequence: first, the center shaft was extracted, then, the retaining nuts were unscrewed, after which the mandrel segments were sequentially removed through the pole holes in the flanges. The metal flanges, wrapped together with the body, remained fixed on the inner surface of the body, ensuring their integration with the composite PS. This solution results in a significant increase in the load-carrying capacity of the structure, as the flanges effectively work together with the composite casing.

Hence, as a result of development of the technology for manufacturing SRM PS from high-strength fibers based on combined reinforcement, the engine casings were manufactured. The technology of manufacturing PS of the SRM casing by winding high-strength carbon fibers impregnated with epoxy resin on a collapsible tooling was successfully tested. Optimum winding angles of ±55° and ±20° were applied for the cylindrical part of the case, providing uniform stress distribution. For hemispherical and cylindrical sections, alternating angles of ±45° and 80° were implemented, which improved resistance to breaking and torsional loads while minimizing deformations under internal pressure. As a result of the technological development with the application of combined reinforcement, engine casings with the necessary characteristics were fabricated.

### 3.4. Research of Composite Power Casing Shell Stability to Internal Pressure and Achievement of Stability up to 10 MPa

**Figure 13:**
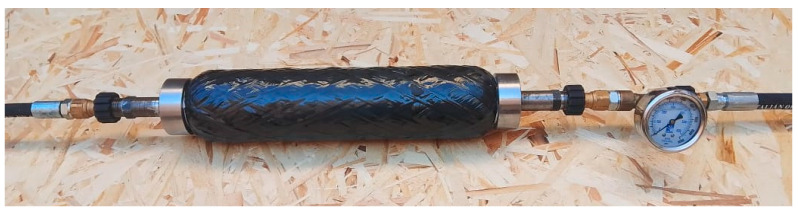
Hydrotesting line.

The strength characteristics of the composite PS under the influence of internal pressure were analyzed. The tests were performed by the method of hydraulic loading at working pressures up to 20 MPa, in order to determine the ultimate resistance to failure. To perform the hydro-tests, a specialized test rig was assembled, including a unit for supplying the working pressure, hermetically connected to the test specimen through screw caps, as well as an outlet unit for pressure fixation (Figure 13).

**Figure 14:**
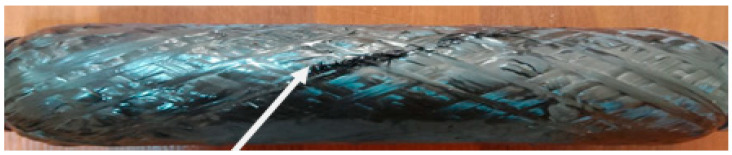
Composite power shell of casing (3 mm wall thickness) after hydrostatic strength test.

The tests covered PS specimens with wall thicknesses of 1.5 mm and 3 mm. The results of the tests determined the following:

- The specimen with wall thickness of 1.5 mm withstood the working pressure up to 10 MPa. At a further increase in the load up to 11 MPa, the cylindrical part of the casing reached failure point.
- The sample with a wall thickness of 3 mm demonstrated resistance to internal pressure up to 18 MPa, while the casing failure took place when this pressure level was exceeded (Figure 14).

As a comparison, maximum load in 10 MPa in similar pressure vessels with winding angles of 90°/0°/±45° were achieved in the paper [2]. The authors of [3] showed that the composite casing wound under 30°/90° exhibited a maximum internal pressure 8 MPa prior to failure.

The results confirmed the capability of composite casings to withstand significant loads with relatively small wall thicknesses, which provides structural weight optimization without significant loss of its strength characteristics.

## 4. Conclusions

Lightweight and strong SRM composite casings with safety factor *n* ≥ 1 at an internal pressure of 10 MPa, fabricated from a polymer composite with a combined winding of carbon fibers impregnated with epoxy resin, have been developed.

A technological tooling for winding has been engineered, the geometry of which corresponds to the inner surface of the case, and provides easy dismantling after curing and has a smooth surface. The main focus of the design is to improve manufacturability, reduce the weight of the tooling, and ensure superior strength of the winding casings.

The winding process was performed utilizing carbon fibers impregnated with epoxy resin on a collapsible mandrel. Winding angles of ±55° and ±20° were used to achieve a uniform stress distribution, especially critical for cylindrical areas. For the hemispherical and cylindrical zones, alternating angles of ±45° and 80° were applied, which improved resistance to breaking and torsional loads and minimized deformation under internal pressures.

Tests on the resistance of the case to internal pressure were carried out by hydrostatic tests at pressures up to 20 MPa. The operational stability at a pressure of 10 MPa for the case with wall thickness of 1.5 mm and 18 for the case of 3 mm was obtained, which confirms the reliability and substantial efficiency of carbon fiber-reinforced polymer composite material. Furthermore, the results propose manufacturing technology for lightweight composite SRM cases.

## Figures and Tables

**Table 1:** Characteristics of ER Etal Inject-T.

Parameters	Properties
Resin part/hardener ratio (weight parts)	100:49.9/100:51.5
Type	Viscous liquid
Epoxy number of the resin part	32–34
Bruckfeld viscosity of the compound at 45 °C	160 ± 50 cPs
Bruckfeld viscosity of the resin part—component A at 25 °C	no more: 460 ± 30 cPs/400 ± 30 cPs
Bruckfeld viscosity of hardener—component B at 25 °C	no more: 200 cPs/450 ± 50 cPs
Martens heat resistance	no more 180 °C
Recommended temperature of components A and B before mixing, °C	40–45/40–45
Tensile strength, MPa	no more 100/120
Static bending strength, MPa	no more 140/150
Glass transition temperature, Tg	199 °C
Curing mode	4 h at 150 °C, +1 h at 180 °C

**Table 2:** Characteristics TCP.

Parameters	Properties
Appearance	Transparent oily liquid
Chemical formula	C_21_H_21_O_4_P; (CH_3_C_6_H_4_O) 3PO
color by APHA	50 max
Acid number, mg KOH/g	0.1 max
Density (пpи 20 °C), g/cm^3^	1.17–1.18
Mass fraction of free phenol, %	0.1 max
Flash point, °C	228 max
Boiling point, °C	280–290
Ignition temperature, °C	249
Self-ignition temperature, °C	369
Mass fraction of volatile substances, %	0.1 max

**Table 3:** Characteristics of carbon roving.

Parameters	Properties
Density, g/cm^3^	1.8
Tensile strength, MPa	4900
Tensile modulus, GPa	230
Relative elongation at break, %	2.1
Number of filaments	12,000 = 12k
Filament diameter, µm	7

## Data Availability

The original contributions presented in this study are included in the article. Further inquiries can be directed at the corresponding author.
